# Retraction: Mouse models of *GNAO1*-associated movement disorder: Allele- and sex-specific differences in phenotypes

**DOI:** 10.1371/journal.pone.0258912

**Published:** 2021-10-14

**Authors:** Huijie Feng, Casandra L. Larrivee, Elena Y. Demireva, Huirong Xie, Jeff R. Leipprandt, Richard R. Neubig

After this article [1] was published, the authors re-sequenced genomic DNA from the *Gnao1*^G203R^ knock-in mouse line and discovered a second mutation in the *Gnao1* locus, present in the founders for the mouse line and all progeny that were analyzed. The two-nucleotide mutation impacts an exon 6 splice acceptor site upstream of the G203R mutation. Whereas G203R is reported to serve as a gain-of-function mutation, the splice acceptor site mutation results in a haploinsufficient loss-of-function allele. In sequence analysis of brain tissue from knock-in mice, all animals positive for the G203R mutation were also found to have the splice acceptor site mutation, and RT-PCR/sequencing analysis revealed that transcripts from this allele were not spliced correctly and did not include the exon with the G203R mutation.

This issue has significant implications for the results and conclusions reported in [1]. Specifically, statements about impacts of the G203R mutation and statements referring to this mouse line as a gain-of-function model and/or a tool to study effects of the human G203R mutation are not supported. In light of this issue, the authors retract this article.

All authors agree with this retraction and apologize for the issues with the published article.
